# Supplementary material for: The Chimeric Nuclease SpRYc Exhibits Highly Variable Performance Across Biological Systems
Source: Int J Mol Sci. 2026 Jan 3;27(1):488. doi: 10.3390/ijms27010488 (PMC12787127; doi:10.3390/ijms27010488)
Supplement: Supplementary file 1 [file ijms-27-00488-s001.zip › ijms-4084157-supplementary.pdf]

Supplementary Information

The chimeric nuclease SpRYc exhibits highly variable performance across biological systems

Irina O. Deriglazova, Mikhail V. Shepelev, Natalia A. Kruglova, Pavel G. Georgiev, and Oksana G. Maksimenko

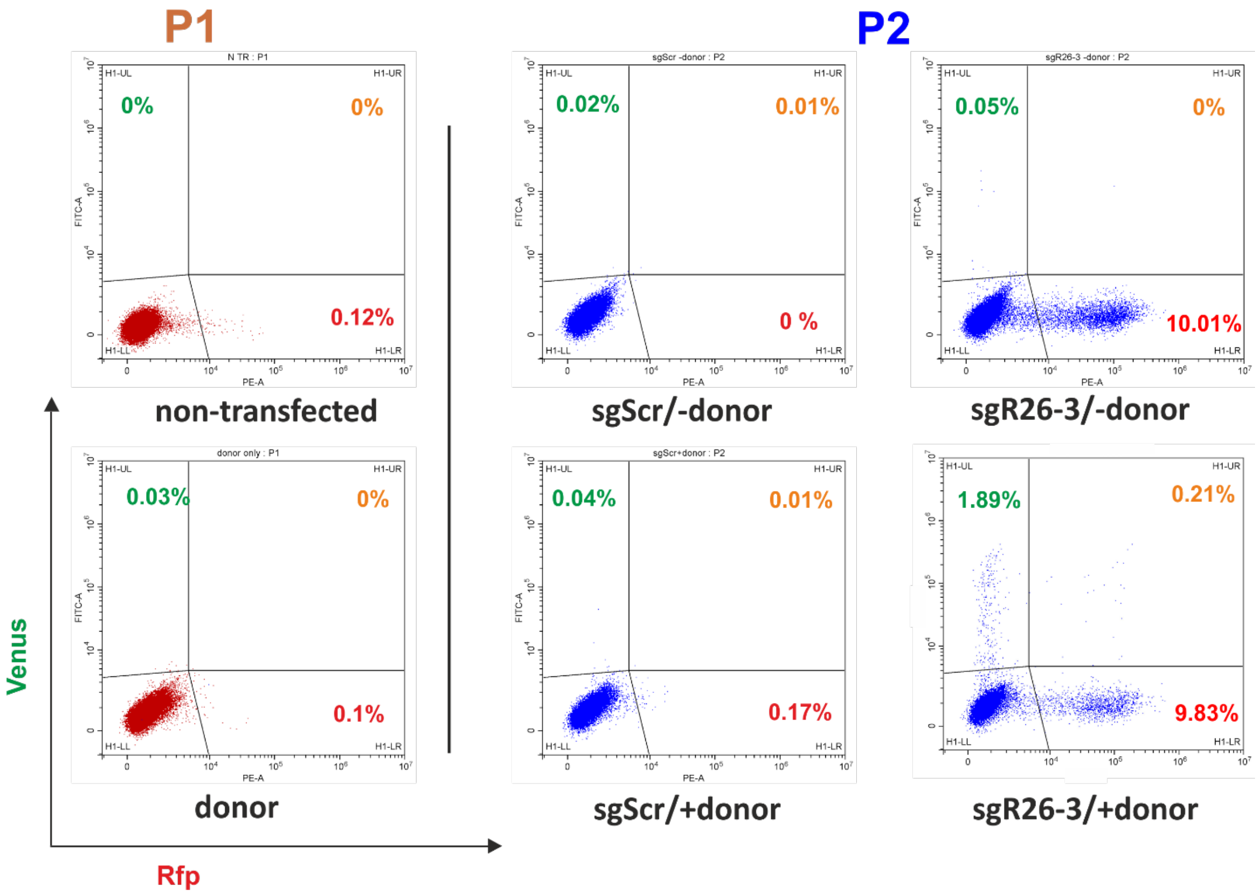

Figure S1. Validation of the TLR5 reporter system. 1 × 10^5 of HEK293-TLR5 clone 9 cells were seeded into wells of 24-well plate in a 0.5 ml of full growth medium without puromycin. Next day, cells were transfected with 600 ng of pDNA mixture (200 ng of pX330-sgR26-3-Cas9-t2a-Ebfp (sgR26-3) or pX330-sgScr-Cas9-t2a-Ebfp (sgScr) + 400 ng of knock-in donor plasmid pTLR-donor-ΔATGΔpolyA) using GenJect-39 (Molecta, #Gen39-500p, Moscow, Russia) transfection reagent. For analysis of genome editing activity three days after transfection cells were trypsinized, washed with growth medium, resuspended in PBS at RT and immediately analyzed by flow cytometry using a CytoFLEX S flow cytometer (Beckman-Coulter, USA). Data were acquired and analyzed by CytExpert 2.0 software (Beckman-Coulter, USA). For analysis of genome editing, first, single cells were gated in P1 gate and then transfected cells co-expressing SpCas9 and Ebfp were gated using gate P2. The resulting KI/KO efficiencies are calculated for P2-gated cells.

Figure S1 shows that NHEJ-mediated editing (10.01%) is detected only when *Rosa26*-targeting sgRNA sgR26-3, but not the control scrambled sgRNA, is co-expressed with SpCas9 (upper right flow cytometry graphic). Knock-in (1.89%) is detected only when donor plasmid pTLR-donor-ΔATGΔpolyA is co-transfected with SpCas9 and sgR26-3. Therefore, we conclude that the TLR5 reporter system in HEK293 cells is functioning as expected and could be used for the evaluation of SpRYc and SpCas9 editing activity.

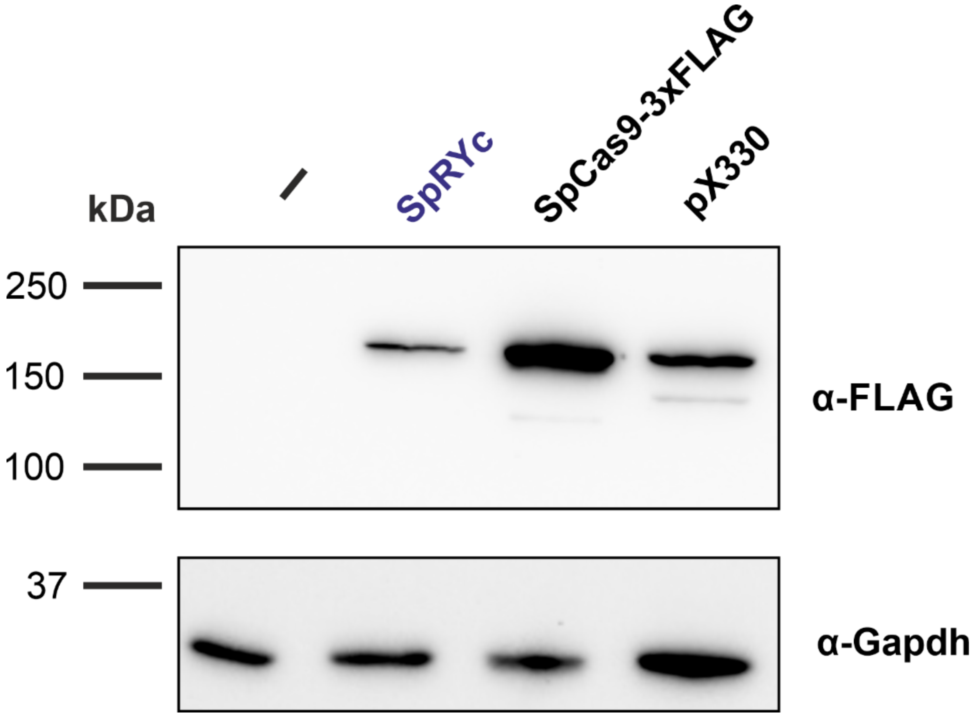

Figure S2. Western-blot analysis of SpRYc expression in HEK293 TLR5 clone 9 cells. Cell lysates were prepared from HEK293-TLR5 clone 9 cells transfected with pCMV-SpRYc, pcDNA3.3-hSpCas9-3×FLAG and pX330-sgR26-1 plasmids, along with a non-transfected control (–). Blots were probed with anti-FLAG and anti-Gapdh (loading control) antibodies.

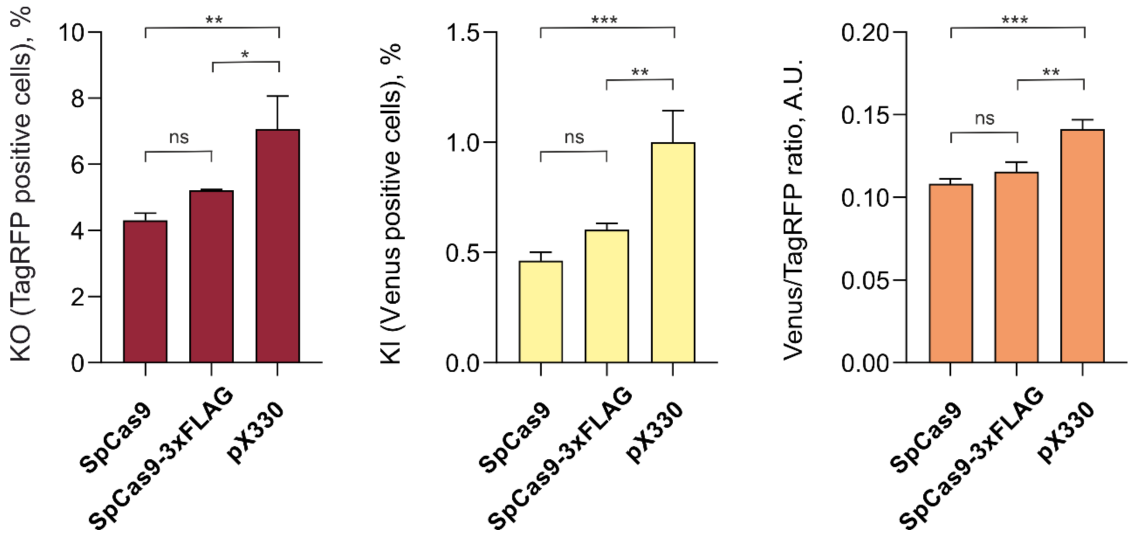

Figure S3. Comparison of SpCas9 and SpCas9-3×FLAG editing activity in HEK293 TLR5 reporter system. 1.25×10^5 of HEK293-TLR5 clone 9 cells were seeded into wells of 24-well plate in a 0.5 ml of full growth medium without puromycin. Next day, cells were transfected with 600 ng of pDNA mixture (200 ng of pcDNA3.3-hSpCas9 (SpCas9) or pcDNA3.3-hSpCas9-3×FLAG (SpCas9-3×FLAG) + 200 ng of pKS-U6-sgR26-1 + 200 ng of pTLR-donor-ΔATGΔpolyA; or with 300 ng of pX330-sgR26-1 + 300 ng of pTLR-donor-ΔATGΔpolyA (pX330)) using GenJect-39 (Molecta, #Gen39-500p, Moscow, Russia) transfection reagent. Cells were analyzed for genome editing activity five days after transfection as described in the Figure S1 legend.

For all genome editing experiments in HEK293 and CEM-R5 T cells we used SpCas9 without epitope tags, therefore we generated SpCas9-3xFLAG and demonstrated that its editing activity is comparable to SpCas9 (Figure S3). KO efficiency (% TagRFP$^+$), KI efficiency (% Venus$^+$), and the KI/KO ratio did not differ between SpCas9 and SpCas9-3×FLAG, whereas pX330 showed significantly higher values for all three metrics compared with both constructs ($p < 0.01$–0.001). Data were analyzed by one-way ANOVA followed by Šídák's multiple comparisons test. Significance: ns, not significant; *, $p < 0.05$; **, $p < 0.01$; ***, $p < 0.001$; ****, $p < 0.0001$. These results clearly indicate, that addition of 3×FLAG epitope at the C-terminus of SpCas9 did not alter its editing efficiency.

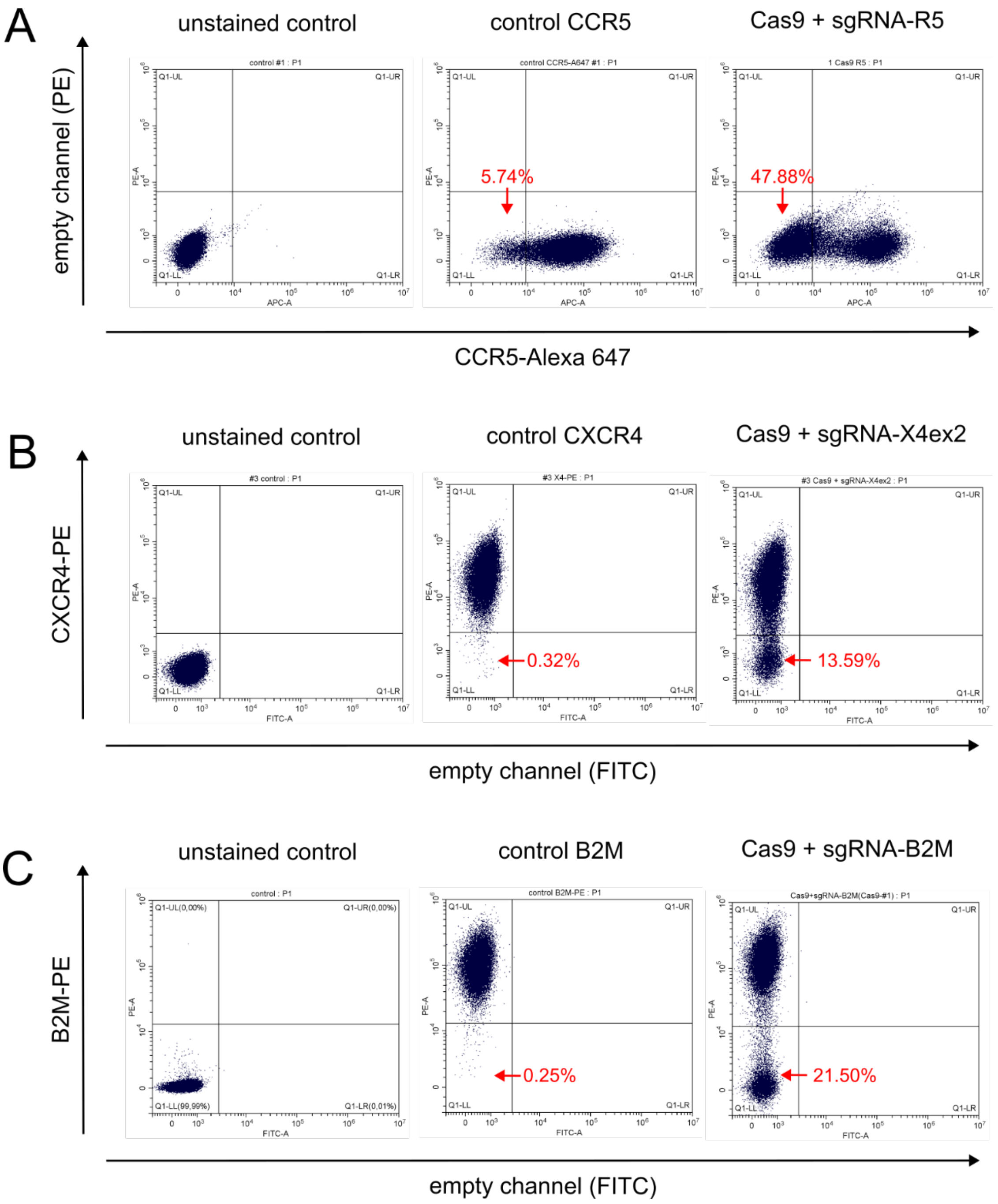

Figure S4. Representative flow cytometry images of edited 293T-CD4-CCR5 clone 19 and CEM-R5 T cells (correspond to Figures 2 and 3). (A) 293T-CD4-CCR5 clone 19 cells were transfected with pcDNA3.3-hSpCas9 or SpRYc plasmids together with one of the respective pKS-U6-sgRNA plasmids. (B, C) CEM-R5 cells were electroporated with pcDNA3.3-hSpCas9 or SpRYc plasmids together with one of the respective pKS-U6-sgRNA plasmids. (A, B, C) The level of knockout was assessed by flow cytometry on day 5 after transfection/electroporation. Cells were stained with antibodies against CCR5, CXCR4, or B2M and analyzed using a CytoFLEX S flow cytometer. Representative images are shown for CCR5 (A), CXCR4 (B), and B2M (C) KO. The percentage of cells in the KO gate is indicated. The level of KO plotted in Figures 2 and 3 is calculated by subtracting this percentage in the control sample from that in the experimental sample.

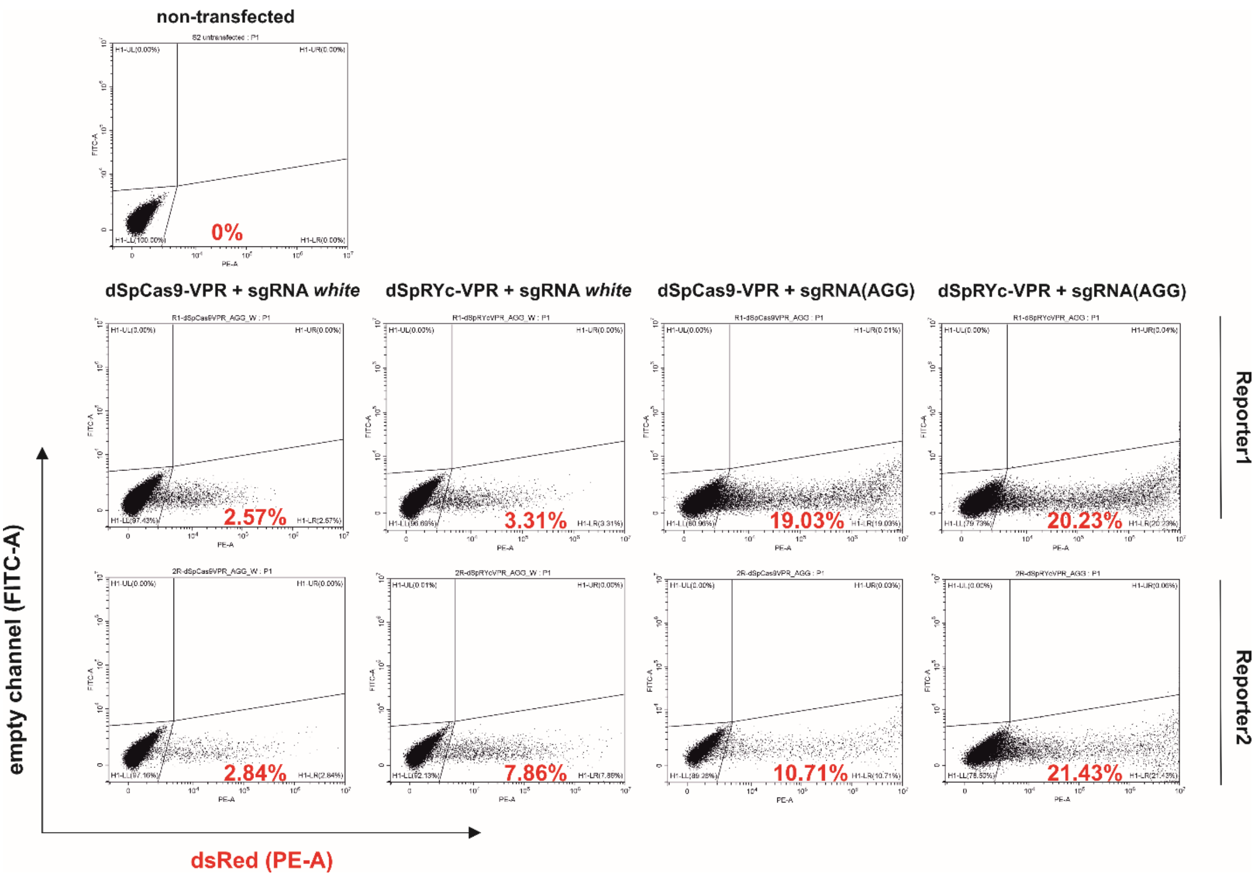

Figure S5. Detection of dsRed-positive Drosophila S2 cells in the PE-A channel in Reporter1 and Reporter2. Flow-cytometry dot plots (PE-A vs FITC-A) show the proportion of dsRed-positive cells under each experimental condition. Non-transfected cells served as the negative control and were used to define the dsRed-negative gate. Compensation between PE-A and FITC-A was adjusted based on the fluorescence profile of the non-transfected sample. Reporter1 and Reporter2 were analyzed using the same gating strategy. Percentages represent the fraction of dsRed-positive events within the designated gate.

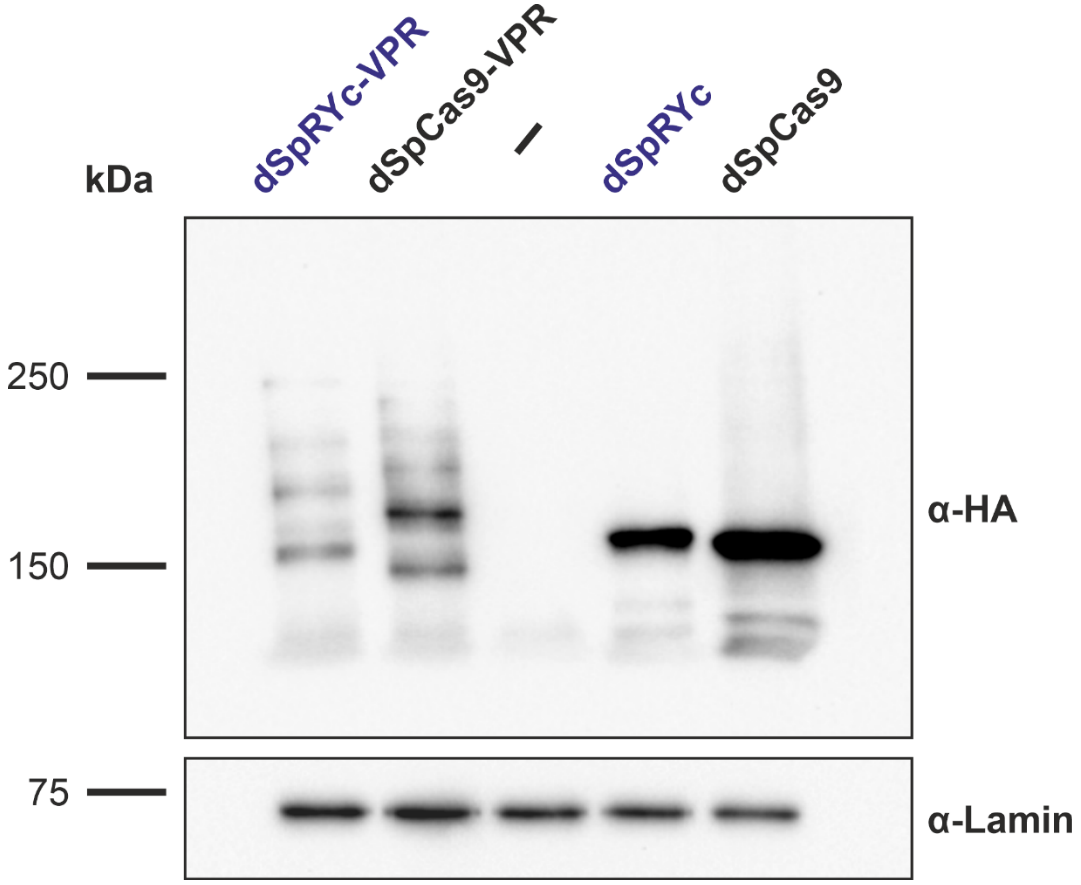

Figure S6. Western blot analysis of dSpCas9-VPR, dSpRYc-VPR, dSpCas9, and dSpRYc in *Drosophila* S2 cells. Cell lysates were prepared from S2 cells transfected with HA-tagged dSpCas9-VPR, dSpRYc-VPR, dSpCas9, and dSpRYc, along with a non-transfected control (–). Blots were probed with anti-HA and anti-lamin (loading control) antibodies.

Table S1. List of guide RNAs.

Experimental model	sgRNA name	Target, 5′->3′	PAM	Oligonucleotides for cloning, 5′->3′
TLR5/Rosa26	sgR26-1	actccagtctttctagaagatgg [29]	TGG	caccgactccagtctttctagaaga<br>aaactcttctagaaagactggagtc
TLR5/Rosa26	sgR26-2	gaagatgggcgggagtcttctgg	TGG	caccgaagatgggcgggagtcttc<br>aaacgaagactcccgcccatcttc
TLR5/Rosa26	sgR26-3	cagtctttctagaagatgggcgg [24]	CGG	caccgcagtctttctagaagatggg<br>aaaccccatcttctagaaagactgc
TLR5/Rosa26	sgR26-4	cgcccatcttctagaaagactgg	TGG	caccgcgcccatcttctagaaagac<br>aaacgtctttctagaagatgggcgc
TLR5/Rosa26	sgScr	gcactaccagagctaactca	-	caccgcactaccagagctaactca<br>aaactgagttacgtctggtagtgc
TLR5/Rosa26	sgSpRYc_1	ctttctagaagatgggcgggagt	AGT	caccgctttctagaagatgggcggg<br>aaaccccgcccatcttctagaaagc
TLR5/Rosa26	sgSpRYc_2	cttctagaaagactggagttgca	GCA	caccgcttctagaaagactggagtt<br>aaacaactccagtctttctagaagc
TLR5/Rosa26	sgSpRYc_3	cccatcttctagaaagactggag	GAG	caccgcccatcttctagaaagactg<br>aaaccagtctttctagaagatgggc
TLR5/Rosa26	sgSpRYc_4	aactccagtctttctagaagatg	ATG	caccgaactccagtctttctagaag<br>aaaccttctagaaagactggagttc
TLR5/Rosa26	sgSpRYc_5	ccgcccatcttctagaaagactg	CTG	caccgccgcccatcttctagaaaga<br>aaactctttctagaagatgggcggc
TLR5/Rosa26	sgSpRYc_6	agcctgcccagaagactcccgcc	GCC	caccgagcctgcccagaagactccc<br>aaacgggagtcttctgggcaggctc
TLR5/Rosa26	sgSpRYc_7	ttctgggcaggcttatatcaagc	AGC	caccgttctgggcaggcttatatca<br>aaactgatataagcctgcccagaac
CXCR4	sgX4ex2	cacttcagataactacaccgagg [31]	AGG	caccgcacttcagataactacaccg<br>aaaccggtgtagttatctgaagtgc
CXCR4	sgX4-agt	tgagcccatttcctcggtgtagt	AGT	caccgtgagcccatttcctcggtgt<br>aaacacaccgaggaaatgggctcac
CXCR4	sgX4-gca	tccattcctttgcctcttttgca	GCA	caccgtccattcctttgcctctttt<br>aaacaaaagaggcaaaggaatggac
CXCR4	sgX4-gag	acacttcagataactacaccgag	GAG	caccgacacttcagataactacacc<br>aaacggtgtagttatctgaagtgtc
CXCR4	sgX4-atg	cagataactacaccgaggaaatg	ATG	caccgcagataactacaccgaggaa<br>aaacttcctcggtgtagttatctgc
CXCR4	sgX4-ctg	catttcctcggtgtagttatctg	CTG	caccgcatttcctcggtgtagttat<br>aaacataactacaccgaggaaatgc
CXCR4	sgX4-gcc	atggagtcatagtcccctgagcc	GCC	caccgatggagtcatagtcccctga<br>aaactcaggggactatgactccatc
B2M	sgB2M-1	gagtagcgcgagcacagctaagg [32]	AGG	caccgagtagcgcgagcacagcta<br>aaactagctgtgctcgcgctactc
B2M	sgB2M-agt	cctccaggccagaaagagagagt	AGT	caccgcctccaggccagaaagagag<br>aaacctctctttctggcctggaggc
B2M	sgB2M-gca	agaaagagagagtagcgcgagca	GCA	caccgagaaagagagagtagcgcga<br>aaactcgcgctactctctctttctc

B2M	sgB2M-gag	tactctctctttctggcctggag	GAG	caccgtactctctctttctggcctg<br>aaaccaggccagaaagagagagtac
B2M	sgB2M-atg	ctgacagcattcgggccgagatg	ATG	caccgctgacagcattcgggccgag<br>aaacctcggcccgaatgctgtcagc
B2M	sgB2M-ctg	gtctcgctccgtggccttagctg	CTG	caccgtctcgctccgtggccttag<br>aaacctaaggccacggagcgagac
B2M	sgB2M-gcc	ccgagatgtctcgctccgtggcc	GCC	caccgccgagatgtctcgctccgtg<br>aaaccacggagcgagacatctcggc
CCR5	sgR5-1	cagcatagtgagcccagaagggg [33]	GGG	caccgcagcatagtgagcccagaag<br>aaaccttctgggctcactatgctgc
CCR5	sgR5-2	tgacatcaattattatacatcgg [34]	CGG	caccgtgacatcaattattatacat<br>aaacatgtataataattgatgtcac
CCR5	sgR5-agt	aggttgagcaggtagatgtcagt	AGT	caccgaggttgagcaggtagatgtc<br>aaacgacatctacctgctcaacctc
CCR5	sgR5-gca	gtcagagatggccaggttgagca	GCA	caccgtcagagatggccaggttga<br>aaactcaacctggccatctctgac
CCR5	sgR5-gag	aggtcagagatggccaggttgag	GAG	caccgaggtcagagatggccaggtt<br>aaacaacctggccatctctgacctc
CCR5	sgR5-atg	tgagcaggtagatgtcagtcatg	ATG	caccgtgagcaggtagatgtcagtc<br>aaacgactgacatctacctgctcac
CCR5	sgR5-ctg	agagcatgactgacatctacctg	CTG	Caccgagagcatgactgacatctac<br>aaacgtagatgtcagtcatgctctc
CCR5	sgR5-gcc	tctgggctcactatgctgccgcc	GCC	caccgtctgggctcactatgctgcc<br>aaacggcagcatagtgagcccagac
CCR5	sgR5-agc	agatgtcagtcatgctcttcagc	AGC	caccgagatgtcagtcatgctcttc<br>aaacgaagagcatgactgacatctc
S2/Reporter 1	7H2RSpy	ttccggtaaggcatgcccaagta	GTA	gtcgttccggtaaggcatgcccaa<br>aaacttgggcatgccttaccggaa
S2/Reporter 1	14H2RSpy	gatcctttccggtaaggcatgcc	GCC	gtcggatcctttccggtaaggcat<br>aaacatgccttaccggaaaggatc
S2/Reporter 1	15H2RSpy	tttccggtaaggcatgcccaagt	AGT	gtcgtttccggtaaggcatgccca<br>aaactgggcatgccttaccggaaa
S2/Reporter 1	16H2RSpy	gtaagatcctttccggtaaggca	GCA	gtcggtaagatcctttccggtaag<br>aaaccttaccggaaaggatcttac
S2/Reporter 1	18H2RSpy	aagatcctttccggtaaggcatg	ATG	gtcgaagatcctttccggtaaggc<br>aaacgccttaccggaaaggatctt
S2/Reporter 1	20H2RSpy	ttgggcatgccttaccggaaagg	AGG	gtcgttgggcatgccttaccggaa<br>aaacttccggtaaggcatgcccaa
S2/Reporter 2	17H2RSpy	ctggaacgatctttgagtgcgag	GAG	gtcgctggaacgatctttgagtgc<br>aaacgcactcaaagatcgttccag
S2/Reporter 2	19H2RSpy	ctttgagtgcgagatcctttctg	CTG	gtcgctttgagtgcgagatccttt<br>aaacaaaggatctcgcactcaaag
S2/Reporter 2	22H2RSpy	actcaaagatcgttccagaaagg	AGG	gtcgactcaaagatcgttccagaa<br>aaacttctggaacgatctttgagt
D.melanogaster/ White	sgW1	ataccattcctgctctttggcgg [25]	CGG	gtcgataccattcctgctctttgg<br>aaacccaaagagcaggaatggtat
D.melanogaster/ White	sgW2	cgccaggcagttgaagaagtgca	GCA	gtcgcgccaggcagttgaagaagt<br>aaacacttcttcaactgcctggcg

D.melanogaster/ White	sgW3	gtatacctcaaatggttgtcgta	GTA	gtcggtatacctcaaatggttgtc aaacgacaaccatttgaggtatac
D.melanogaster/ White	sgW4	cgccggttatcataccattcctg	CTG	gtcgcgccggttatcataccattc aaacgaatggtatgataaccggcg
D.melanogaster/ White	sgW5	cagacagcgccatcgaggtcgag	GAG	gtcgcagacagcgccatcgaggtc aaacgacctcgatggcgctgtctg
S2/Reporter 1 & 2 *D.melanogaster/ White*	sgW6	acccacagacagcgccatcgagg	AGG	gtcgacccacagacagcgccatcg aaaccgatggcgctgtctgtgggt

Table S2. Plasmid vectors used in this study.

**Plasmid name**	**Description**	**Source**
pBluescriptSKII (+)	General cloning vector	Stratagene, San Diego, USA
SpRYc	Co-expression of SpRYc and Egfp in mammalian cells (used as a template for PCR amplification of the SpRYc fragment)	Addgene #175575, [18]
pCMV-SpRYc	Expression of SpRYc in mammalian cells	This study
hCas9	Expression of SpCas9 in mammalian cells	Addgene #41815, [39]
pcDNA3.3-hSpCas9-3×FLAG	Expression of SpCas9-3×FLAG in mammalian cells	This study
pKS-U6-gRNA-BB	BbsI-mediated cloning of sgRNAs for SpRYc and SpCas9	[31]
pSK-U6:3Dmel	BbsI-mediated cloning of sgRNAs for SpRYc and SpCas9	This study
pTLR-donor-ΔATGΔpolyA	Donor plasmid for repair of Venus sequence in TLR5 reporter system	[24]
pU6-(BbsI)_CBh-Cas9-T2A-BFP	Co-expression of SpCas9, sgRNA and BFP	Addgene #64323, [29]

pX330-U6-Chimeric_BB-CBh-hSpCas9	Co-expression of SpCas9 and sgRNA	Addgene #42230, [40]
pX330-sgR26-1	Co-expression of SpCas9 and sgR26-1 in TLR5 reporter system	This study
pX330-sgR26-3-Cas9-t2a-Ebfp	Co-expression of SpCas9, sgR26-3 and Ebfp in TLR5 reporter system	This study
pX330-sgScr-Cas9-t2a-Ebfp	Co-expression of SpCas9, sgScr and Ebfp in TLR5 reporter system	This study
pAct:dCas9-VPR	Expression of dCas9-VPR in *D.melanogaster* (used as a template for PCR amplification of the VPR fragment)	Addgene #78898, [36]
pAc5.1	Plasmid for expression of cDNA in Drosophila cells under control of Act5c promoter	Invitrogen, USA
pAct5c-SpRYc	Expression of SpRYc in *D.melanogaster*	This study
pDsRed-attP	Template for PCR amplification of 3xP3, minimal Hsp70 promoter and dsRed	Addgene #51019, [37]
pAct-Cas9	Expression of Cas9 in *D.melanogaster*	Addgene #62209, [38]
pCFD4-U6:1_U6:3tandemgRNAs	The plasmid from which the sequence of U6:3, two BbsI recognition sites, and the Cas9 scaffold RNA was cloned	Addgene #49411, [38]
yiless5×Pi-pUbi	Vector for construction of expression plasmid. It contains the promoter of Ubiquitin-63E gene and polyadenylation signal	[35]
yiless5×Pi-pUbi-dSpRYc-FLAG	Construction of dSpRYc-VPR expression plasmid for Drosophila	This study

yiless5×Pi-pUbi-dSpCas9	Construction of dSpCas9-VPR expression plasmid for Drosophila	This study
yiless5×Pi-pUbi-dSpCas9-HA	Construction of dSpCas9-VPR expression plasmid for Drosophila	This study
yiless5×Pi-pUbi-dSpRYc-FLAG-HA-VPR	Expression of dSpRYc-VPR under the control of *Drosophila* Ubiquitin-63E promoter. Mutations: D10A, H849A	This study
yiless5×Pi-pUbi-dSpCas9-HA-VPR	Expression of dSpCas9-VPR under the control of *Drosophila* Ubiquitin-63E promoter	This study
pSK-attB-3×PE-Hsp70dsRed	Construction of Reporter plasmids (pSK-Reporter1 and pSK-Reporter2)	This study
pSK-attB-3×PE-x1Trg1-Hsp70dsRed	Reporter 1 with 1x repeat sequence	This study
pSK-attB-3×PE-x2Trg1-Hsp70dsRed	Reporter 1 with 2x repeat sequences	This study
pSK-Reporter1 (pSK-attB-3×PE-×4Trg1-Hsp70dsRed)	Reporter 1 with 4x repeat sequences	This study
pSK-attB-3×PE-×1Trg4-Hsp70dsRed	Reporter 2 with 1x repeat sequence	This study
pSK-attB-3×PE-×2Trg4-Hsp70dsRed	Reporter 2 with 2x repeat sequences	This study
pSK-Reporter2/ pSK-attB-3×PE-×4Trg4-Hsp70dsRed	Reporter 2 with 4x repeat sequences	This study

Table S3. Oligonucleotides used for cloning.

Oligonucleotide	Sequence, 5′->3′	Description
SpRYcKpn_d	ggcggtaccatggagaagaaatactcc	Cloning of SpRYc into the pAc5.1 plasmid vector
flagSalNot_r	tttgcggccgctttagtcgactcccttgtcatcgtcatcc	Cloning of SpRYc into the pAc5.1 plasmid vector Cloning of dSpRYc into the yiless5×Pi-pUbi-dSpRYc-FLAG-HA-VPR
CasD10A_d	cctggccatcggaaccaacag	Used to introduce the D10A mutation in SpRYc
CasD10A_r	ggttccgatggccaggcc	Used to introduce the D10A mutation in SpRYc
CasH849A_d	gtggaccggatcgtgccc	Used to introduce the H849A mutation in SpRYc
CasH849A_r	gcacgatggcgtccacatcg	Used to introduce the H849A mutation in SpRYc
HAtagSal_d	aaagtcgacggatcctacccatacgatgttccaga	Used to amplify 3×HA tag fragment for fusion with VPR fragment and cloning into the yiless5×Pi-pUbi-dSpRYc-FLAG-HA-VPR
HAtagNhe_r	gggggatccgctagcggcatagtcgggga	Used to amplify 3×HA tag fragment for fusion with VPR fragment
NheVpr_d	gctagcggatcccccaagaagaagaggaaggt	Used to amplify VPR fragment for fusion with 3×HA tag fragment
VPR_NotI_fustSV40_r	cGGTACCgcggccgctcaaaacagagatgtgt	Used to amplify VPR fragment for fusion with 3×HA tag fragment and cloning to the yiless5×Pi-pUbi-dSpRYc-FLAG-HA-VPR
SpeN-SpRYc_d	aaaactagtcatggagaagaaatactccatcggc	Cloning of dSpRYc into the yiless5×Pi-pUbi-dSpRYc-FLAG-HA-VPR
NhedC9_d	aaagctagcaatggacaagaagtactccattggg	Cloning of dSpCas9 into the yiless5×Pi-pUbi-dSpCas9-HA-VPR
SalNcodC9_r	tttccatgggtcgacgctgtctccaccgagc	Cloning of dSpCas9 into the yiless5×Pi-pUbi-dSpCas9-HA-VPR
SalIattBmBsa_d	aaagtcgacacagtctcgaagccg	Used to amplify attB fragment for fusion with 3×P3 fragment and cloning into the pBluSK
attBfus3×PE_r	gatccccgtcgagaacccgct	Used to amplify attB fragment for fusion with 3×P3 fragment
3×PE_d	gttctcgacggggatctaattcaattagag	Used to amplify 3×P3 fragment for fusion with attB fragment and minimal promoter Hsp70, dsRed and SV40 polyadenylation signal
3×PE-BHI_r	ggttcgaatctagaatttggatcctaattgaattagc	Used to amplify 3×P3 fragment for fusion with attB fragment and minimal promoter Hsp70, dsRed and SV40 polyadenylation signal

Hsp70m_d	ccaaattctagattcgaaccctcgacc	Used to amplify minimal promoter Hsp70, dsRed and SV40 polyadenylation signal for fusion with 3×P3
tsv40-PstI-Spe_r	tttactagttctgcagaagatacattgatgagtttgg	Used to amplify minimal promoter Hsp70, dsRed and SV40 polyadenylation signal for fusion with 3×P3 fragment and cloning into the pBluSK
dC12Trg1_d	gatcctttccggtaaggcatgcccaagtaagatctaaat	Cloning of 'repeat sequences' in pSK-Reporter1
dC12Trg1_r	ctagatttagatcttacttgggcatgccttaccggaaag	Cloning of 'repeat sequences' in pSK-Reporter1
dC12Trg4_d	gatcctttctggaacgatctttgagtgcgagatctaaat	Cloning of 'repeat sequences' in pSK-Reporter2
dC12Trg4_r	ctagatttagatctcgcactcaaagatcgttccagaaag	Cloning of 'repeat sequences' in pSK-Reporter2
casF_RI	aaataagcgaattctccaaaag	Cloning of 3×FLAG tag to the C-terminus of SpCas9
sv40nls_R	ccgctcgacaccttcctcttcttc	Cloning of 3×FLAG tag to the C-terminus of SpCas9
Flag_spryc_f	gaaggtgtcgagcggaggcgacta	Cloning of 3×FLAG tag to the C-terminus of SpCas9
Flag_Age	aaaccggtttagtcgactcccttgtc	Cloning of 3×FLAG tag to the C-terminus of SpCas9

Table S4. Oligonucleotides used for ChIP-qPCR.

Oligonucleotide	Sequence, 5′->3′
Rpl32_d	gttcgatccgtaaccgatgt
Rpl32_r	ccagtcggatcgatatgctaa
dsRed_d	ggacgtcatcaaggagttcat
dsRed_r	ttggtcaccttcagcttgg
W_ed_d	cgtggtgttcgacgatgtg
W_ed_r	ttacgccaacgagggtct
